# Schizophrenia and potentially preventable hospitalizations in the United States: a retrospective cross-sectional study

**DOI:** 10.1186/1471-244X-13-37

**Published:** 2013-01-25

**Authors:** Elizabeth Khaykin Cahoon, Emma E McGinty, Daniel E Ford, Gail L Daumit

**Affiliations:** 1 Department of Mental Health, Johns Hopkins Bloomberg School of Public Health, Baltimore, MD, USA; 2 Department of Health Policy and Management, Johns Hopkins Bloomberg School of Public Health, Baltimore, MD, USA; 3 Johns Hopkins Medical Institutions, Division of General Internal Medicine, Welch Center for Prevention, Epidemiology and Clinical Research, 2024 East Monument Street, Room 2-513, Baltimore, MD, USA

**Keywords:** Schizophrenia, Ambulatory care-sensitive condition, Preventable hospitalization, Primary care, United States

## Abstract

**Background:**

Persons with schizophrenia may face barriers to high quality primary care due to communication difficulties, cognitive impairment, lack of social support, and fragmentation of healthcare delivery services. As a result, this group may be at high risk for ambulatory care sensitive (ACS) hospitalizations, defined as hospitalizations potentially preventable by timely primary care. The goal of this study was to determine if schizophrenia is associated with overall, acute, and chronic ACS hospitalizations in the United States (US).

**Methods:**

We conducted a retrospective cross-sectional study. Hospitalization data for the US were obtained from the Nationwide Inpatient Sample for years 2003–2008. We examined 15,275,337 medical and surgical discharges for adults aged 18–64, 182,423 of which had a secondary diagnosis of schizophrenia. ACS hospitalizations were measured using the Agency for Healthcare Research and Quality’s Prevention Quality Indicators (PQIs). We developed logistic regression models to obtain nationally-weighted odds ratios (OR) for ACS hospitalizations, comparing those with and without a secondary diagnosis of schizophrenia after adjusting for patient, hospitalization, and hospital characteristics.

**Results:**

Schizophrenia was associated with increased odds of hospitalization for acute ACS conditions (OR = 1.34; 95% CI: 1.31, 1.38), as well as for chronic ACS conditions characterized by short-term exacerbations. Schizophrenia was associated with decreased odds of hospitalization for diabetes mellitus long-term complications and diabetes-related lower extremity amputation, conditions characterized by long-term deterioration.

**Conclusions:**

Additional research is needed to determine which individual and health systems factors contribute to the increased odds of hospitalization for acute PQIs in schizophrenia.

## Background

Persons with schizophrenia have higher rates of medical comorbidity and mortality compared to the overall population. The literature documents high rates of HIV infection, [1] respiratory illness, [2] obesity [3-5] and diabetes [6,7] in this group, and cardiovascular mortality is twice as high in persons with schizophrenia compared to the overall United States (US) population [8,9]. Poverty, [10] lack of physical activity, [11,12] poor diet, [13,14] high rates of smoking, [13,15] and side effects of antipsychotic medication, such as weight gain, [16,17] likely contribute to the increased prevalence of medical comorbidities and premature mortality among persons with schizophrenia.

Given the high burden of medical conditions among persons with schizophrenia, access to high quality primary care is important for effective disease management. However, persons with schizophrenia may experience inadequate or delayed access to primary care in the US. A 2003 study reported that veterans with schizophrenia were less likely to have a primary care visit than veterans without schizophrenia [18]. A study of persons with serious mental illness receiving care at a community mental health center found that 24% had not had a primary care visit in the past 6 months, and 63% of patients with a primary care provider were unable to identify that provider by name [19]. Studies of the overall US population [20] and psychiatric outpatients, [21] respectively, show that while persons with schizophrenia accessed primary care as or more frequently than the overall population, they were still more likely to report delays in needed care.

Persons with schizophrenia may also experience poor quality primary care. A 2009 review of 27 studies comparing quality of medical care in persons with and without mental illness found that persons with mental illness experienced worse quality of care than persons without mental illness in 70% of studies [22]. For example, two studies of quality of care for diabetes in privately insured adults [23] and veterans, [24] respectively, reported that persons with mental illness were less likely to receive a hemoglobin A1c test, [23] cholesterol screening, [23] foot sensory examination, [24] and retina examination [24] than persons without mental illness.

At the individual level, communication difficulties, [25] cognitive impairment, [26] lack of social support, [27] and a tendency for physicians to perceive physical complaints as psychosomatic in patients with schizophrenia [28] may affect primary care access and quality among this group. At the system level, fragmentation of behavioral and medical health delivery services may be a barrier to timely primary care for persons with schizophrenia [29]. Behavioral and medical services are often provided in systems with separate providers, locations, and sources of funding, factors which potentially impede care coordination across systems.

Poor access to high quality primary care among persons with schizophrenia may lead to ambulatory care-sensitive (ACS), hospitalizations, defined as hospitalizations potentially preventable by timely and effective outpatient care [30]. Ambulatory care sensitive hospitalizations include admissions for conditions such as hypertension and diabetes [31]. High rates of ACS hospitalizations signal potential areas of poor access to or quality of primary care that warrant further investigation at the health system level [31]. Multiple studies have confirmed that high rates of ACS hospitalizations are associated with poor access to primary care in the US population [32-34]. Furthermore, studies suggest that ACS hospitalization rates are heightened among disadvantaged population groups, including racial and ethnic minorities [35]. To date, however, little is known about the association between schizophrenia and ACS hospitalizations in the US.

Studies conducted in Taiwan [36] and Australia [37] found that persons with serious mental illness were at increased risk of ACS hospitalization. However, differences in disease prevalence, access to primary care, and social and cultural treatment of persons with schizophrenia limit the generalizability of these findings to the US. To our knowledge, only one study of ACS hospitalizations among persons with mental illness has been conducted in the US. A study using New York State hospital discharge records in 2004 found that schizophrenia was associated with an 82% increased likelihood of overall ACS hospitalization in adults aged 20–64 years [38].

To date, we lack a comprehensive picture of the association between schizophrenia and specific types of ACS hospitalizations across the entire US healthcare system. Barriers to high quality primary care may differ for acute and chronic ACS conditions among persons with schizophrenia, making it important to describe the likelihood of acute and chronic ACS hospitalizations, respectively, in this population. To our knowledge, no study to date has examined the association between schizophrenia and specific types of ACS hospitalizations in the US.

## Methods

### Data source

Hospital discharge data were obtained from the Nationwide Inpatient Sample (NIS), the largest all-payer inpatient care database in the US, comprised of about 1,000 hospitals across 35 states, with stratified sampling designed to represent all US census regions. The NIS is stratified by geographic region (Northeast, Midwest, West, or South), location (urban or rural), teaching status (teaching or non-teaching), ownership (public, private not-for-profit, or private investor-owned), and bed size (small, medium, or large). It contains data on age, sex, race, length of stay, and ICD-9-CM codes for up to 15 diagnoses and 15 procedures. Discharges are linked to information on hospital characteristics from the American Hospital Association’s Annual Survey Database.

### Measures

The outcomes of interest were ACS hospitalizations. Based upon work by Billings [30], the Agency for Healthcare Quality and Research (AHRQ) developed a set of indicators called the Prevention Quality Indicators (PQIs) to identify ACS hospitalizations. We used AHRQ’s PQI software [31] to create indicators of four acute and nine chronic ACS hospitalizations. Acute ACS hospitalizations included those with primary diagnoses of bacterial pneumonia, urinary tract infection, dehydration and perforated appendix [31]. Chronic ACS hospitalizations included hospitalizations with primary diagnoses of congestive heart failure (CHF), chronic obstructive pulmonary disease (COPD), asthma, diabetes mellitus long and short term complications, hypertension, angina without procedure, diabetes related lower-extremity amputation, and uncontrolled diabetes [31]. Composite overall, acute, and chronic ACS hospitalizations were also measured.

The primary exposure of interest was secondary diagnosis of schizophrenia (ICD-9-CM code 295). Potential confounders were included in the model based on previous research in this field, differences found in Table 1, and so those that remained significant after adjusting for other covariates for the vast majority of PQI outcomes. Measured covariates included age, sex, race, insurance type (uninsured hospitalizations are included in the ‘other’ category), median household income quartile for patient zip-code, hospital region, hospital teaching status, urban/rural designation, hospital bed size, hospital volume, admission source, hospitalization characteristics (elective vs. non-elective, medical versus surgical, against medical advice, and death in hospital) and 12 medical co-morbidities (Table 1). As race data was missing for nine states, missing race was included as a unique category in the race variable. Measures of medical comorbidity were generated using AHRQ’s Clinical Classifications Software [39].

**Table 1:** **Characteristics of hospitalizations for patients with and without schizophrenia among medical and surgical hospitalizations**, **Nationwide Inpatient Sample 2003 to 2008**^ab^

		**With**	**Without**
		**schizophrenia**	**schizophrenia**
**Characteristic**		**(n** **=** **182,423)**	**(n** **=** **15,092,914)**
Median age, years		49.4	48.8
(inter-quartile range)		41.7-56.1	39.0-56.6
Sex, %			
	Male	55.0	47.1
	Female	45.0	52.9
Race, %			
	White	43.6	49.0
	Black	21.9	13.0
	Hispanic	6.7	8.4
	Other or missing	27.8	29.7
Insurance type, %			
	Medicare	47.4	15.5
	Medicaid	35.2	15.8
	Private	8.8	52.6
	Other	8.6	16.2
Median household income			
quartile for patient ZIP code, % ^c^			
	Quartile 1 (lowest)	42.3	29.8
	Quartile 2	26.4	26.1
	Quartile 3	19.0	23.3
	Quartile 4 (highest)	12.3	20.8
Region of country for hospital, %			
	Northeast	22.5	19.4
	Midwest	24.7	22.6
	South	35.7	40.5
	West	17.1	17.5
Teaching hospital (vs. non-teaching)		49.6	47.6
Urban hospital location (vs. rural)		88.0	87.5
Hospital bed size ^d^			
	Small	12	11.5
	Medium	25.3	24.3
	Large	62.7	64.2
Hospital volume, % ^e^			
	Quartile 1 (lowest)	27.7	24.9
	Quartile 2	26.1	25.0
	Quartile 3	23.3	25.0
	Quartile 4 (highest)	22.9	25.0
Admission source, %			
	Emergency room	76.7	57.2
	Court or law enforcement	0.3	0.1
	Routine or other	23.0	42.7
Hospitalization characteristics, %			
	Elective admission (vs. non-elective) ^f^	10.7	28.0
	Medical admission (vs. surgical)	71.3	54.6
	Against medical advice	3.5	1.6
	Death in hospital	1.6	1.4
Comorbid illness, %			
	Congestive heart failure	6.4	3.8
	Hypertension	40.2	36.6
	Chronic obstructive pulmonary disease	25.1	14.0
	Liver disease	4.4	3.1
	Rheumatoid arthritis or collagen vascular diseases	1.0	2.0
	Diabetes mellitus or obesity	31.2	24.6
	Any malignancy	2.2	3.8
	HIV/AIDS	0.9	0.5
	Deficiency anemia	13.4	10.1
	Alcohol abuse	9.6	5.3
	Drug abuse	11.6	4.2
	Any substance abuse	17.4	8.3
Median length of stay, days		3.1	2.3
(inter-quartile range)		1.5-5.9	1.1-4.4

^a ^Results are nationally weighted and account for stratified sampling.

^b ^All characteristics are significantly different except urban/rural and bed size for 18–64; urban/rural, liver for 65+ group.

^c ^Quartile cut-offs vary from year to year. Please see documentation at http://www.hcup-us.ahrq.gov.

^d ^Cutoff points for bed size were made so that approximately one-third of the hospitals in a given region, location, and teaching status combination would fall within each bed size category.

^e ^Cut-offs for hospital volume quartiles in all hospitalizations are 8722, 16746, and 26080; among 18–64 they are 9676, 18033, and 27707; and among 65+ they are 7870, 15494, and 24577.

^f^ Non-elective admissions include emergency, urgent, newborn, delivery, and trauma.

### Study sample

The study sample included hospital discharges with a primary medical or surgical diagnosis for years 2003–2008 among adults aged 18–64 years. As universal Medicare coverage for persons aged 65 years and older may affect the likelihood of ACS hospitalization among older adults, this age group was excluded from analysis. Hospitalizations included in the sample reflect the risk set for a particular ACS hospitalization, as defined by AHRQ. For example, the risk set for perforated appendix includes hospitalizations with a primary diagnosis of appendicitis [40]. To protect patient confidentiality, the NIS does not have identifiers to link hospitalizations to unique individuals. The Johns Hopkins Institutional Review Board deemed that this study met criteria for exemption.

### Statistical analysis

Differences in nationally weighted proportions of patient, hospitalization, and hospital characteristics among hospitalizations with and without secondary schizophrenia diagnosis were tested using Wilcoxon rank sum tests for continuous variables and chi-square tests for categorical variables (Table 1). We also determined the nationally weighted proportions of ACS hospitalizations stratified by age group and secondary diagnosis of schizophrenia (Table 2).

**Table 2:** **Ambulatory care sensitive** (**ACS**) **hospitalizations among medical and surgical hospitalizations for patients with and without schizophrenia**, **Nationwide Inpatient Sample 2003 to 2008**^a^

	**Percent (95% CI)**		**Odds ratio (95% CI)**
	**With**	**Without**	
**Indicator**	**schizophrenia**	**schizophrenia**	
**Overall ACS hospitalizations**:	20.88 (20.55-21.22)	13.13 (12.89-13.37)	1.75 (1.71-1.79)
**Acute ACS hospitalizations**:			
Bacterial pneumonia	4.87 (4.71-5.03)	1.21 (4.71-5.03)	1.94 (1.87-2.00)
Urinary tract infection	1.65 (1.57-1.73)	1.24 (1.22-1.27)	1.34 (1.27-1.40)
Dehydration	2.57 (2.53-2.62)	0.73 (0.71-0.75)	1.67 (1.58-1.76)
Perforated appendix^b^	51.41 (47.47-55.06)	26.71 (26.31-27.12)	2.90 (2.51-3.36)
Composite acute ACS^c^	7.57 (7.39-7.76)	4.43 (4.36-4.50)	1.77 (1.72-1.82)
**Chronic ACS hospitalizations**:			
CHF	2.99 (2.85-3.13)	2.30 (2.24-2.37)	1.31 (1.25-1.37)
COPD	3.25 (3.10-3.41)	1.37 (1.33-1.41)	2.42 (2.31-2.53)
Asthma	2.10 (2.00-2.21)	1.58 (1.53-1.63)	1.34 (1.28-1.40)
DM long-term complication	1.70 (1.61-1.78)	1.29 (1.26-1.32)	1.32 (1.26-1.39)
DM short-term complication	1.49 (1.41-1.58)	0.92 (0.90-0.94)	1.63 (1.54-1.73)
Hypertension	0.61 (0.56-0.66)	0.58 (0.56-0.60)	1.05 (0.98-1.13)
Angina without procedure	0.28 (0.25-0.31)	0.41 (0.39-0.43)	0.68 (0.61-0.75)
Lower-extremity amputation^d^	1.02 (0.92-1.12)	1.65 (1.61-1.69)	0.61 (0.55-0.67)
Uncontrolled diabetes	0.84 (0.78-0.91)	0.27 (0.25-0.28)	3.15 (2.95-3.36)
Composite chronic ACS	13.31 (13.02-13.6)	8.70 (8.51-8.89)	1.61 (1.57-1.65)

Abbreviations: ACS, ambulatory care sensitive; CHF, congestive heart failure; COPD, chronic obstructive pulmonary disease; DM, diabetes mellitus; CI, confidence interval.

^a ^Results are nationally weighted and account for stratified sampling.

^b ^Among admissions for appendicitis.

^c ^Does not include admissions for perforated appendix.

^d ^Among admissions for diabetes.

**Figure 1:**
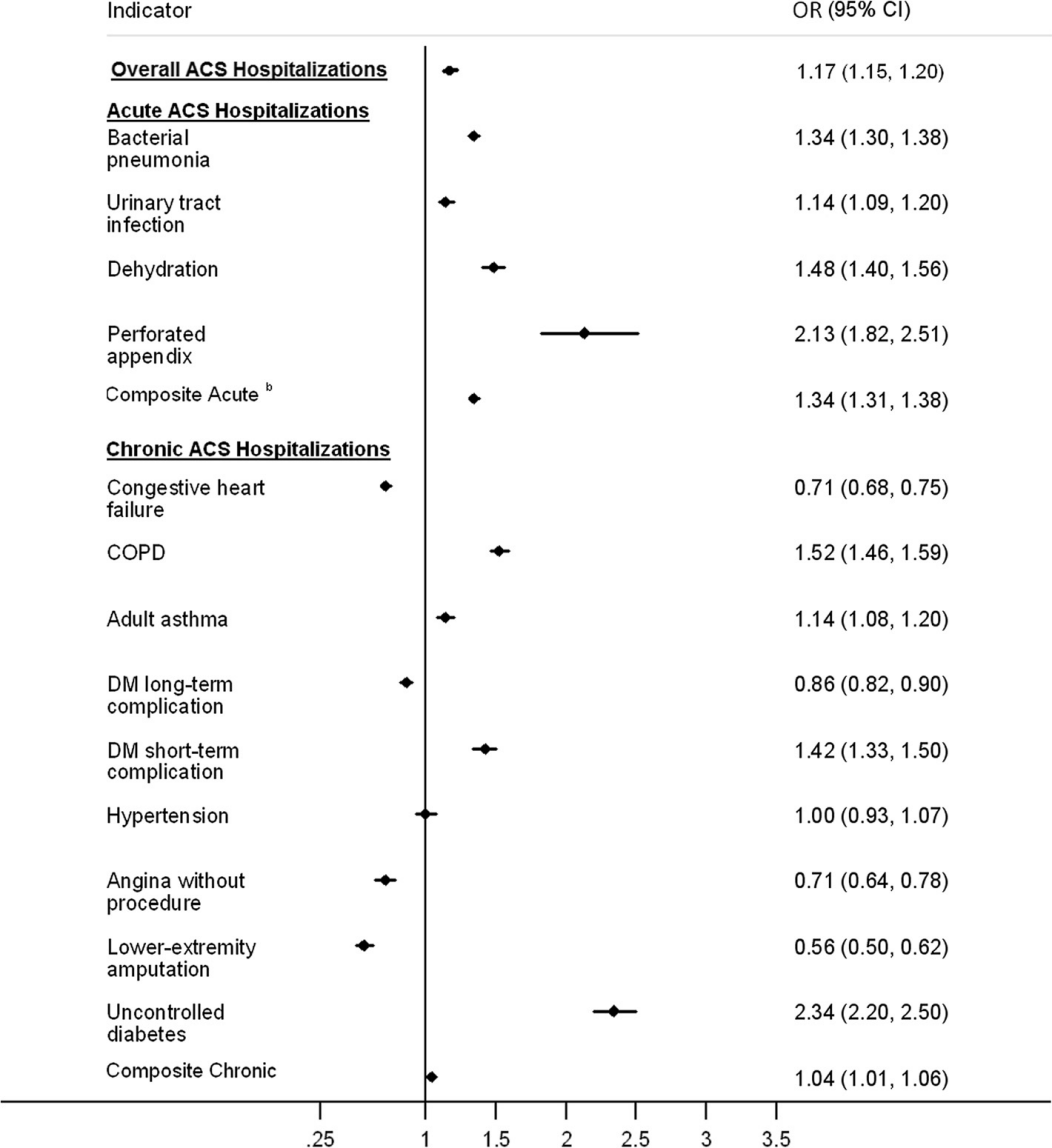
**Odds ratios of ambulatory care sensitive hospitalizations (ACS) comparing patients with and without schizophrenia, by age group, Nationwide Inpatient Sample 2003**–**2008.**

To test the association between schizophrenia and ACS hospitalizations, we developed logistic regression models to obtain odds ratios (ORs) comparing the likelihood of hospitalization for an ACS condition in admissions with versus without secondary diagnosis of schizophrenia. Odds ratios were calculated for the four acute and nine chronic ACS hospitalizations identified by AHRQ, as well as for composite overall, acute, and chronic ACS hospitalizations. Models adjusted for the covariates listed above (Figure 1). Analyses accounted for the NIS sampling scheme by nesting within stratum and hospital and incorporated discharge weights. While analyzing subpopulations, we included all individuals outside the subpopulation to create the correct standard errors. Since the level of observation was hospitalization and multiple hospitalizations per patient were likely, survey design based variance estimators were used to account for within-respondent correlation. Since a year interaction term did not affect results, years 2003 to 2008 were combined. All tests were two-sided and p-values were considered significant at the 0.05 level. Analyses were conducted with SAS 9.2 and SAS-Callable SUDAAN 10 software (Research Triangle Institute).

## Results

### Characteristics of hospitalizations with and without schizophrenia

The study sample included 182,423 and 15,092,914 medical and surgical hospitalizations for adults with and without schizophrenia, respectively (Table 1). Hospitalizations with a secondary diagnosis of schizophrenia were more likely to be non-white, have a low-income zip code of residence, be discharged from a teaching or low-volume hospital, and be emergency, medical (versus surgical), or non-elective admissions. Over 82% of admissions had either Medicaid or Medicare (among adults aged 18–64 years, Medicare coverage indicates receipt of Social Security Disability Insurance). Hospitalizations with schizophrenia had higher proportions of several co-morbid medical conditions, including diabetes mellitus and substance abuse.

### Association between schizophrenia and ACS hospitalizations

Table 2 shows the proportions of medical and surgical hospitalizations accounted for by ACS hospitalizations. Unadjusted ORs of overall, acute, and chronic ACS hospitalizations were higher for admissions with secondary diagnosis of schizophrenia compared to those without. Unadjusted ORs of all four individual acute and six of the nine chronic ACS conditions measured, including CHF, COPD, asthma, diabetes mellitus long and short-term complications, and uncontrolled diabetes were higher for hospitalizations with versus without secondary diagnosis of schizophrenia. The proportion of ACS hospitalizations among medical and surgical hospitalizations was highest for perforated appendix, reflecting this indicator’s small risk set that includes only admissions with appendicitis.

In a nationwide US sample, schizophrenia was associated with 17% increased odds of any ACS hospitalization (Figure 1). Schizophrenia was associated with increased odds of acute ACS hospitalization (OR 1.34, 95% CI 1.31-1.38). Schizophrenia was also associated with increased odds of chronic ACS hospitalization (OR 1.04, 95% CI 1.01-1.06), however this result appears to be driven by the heightened odds of hospitalization for four chronic ACS conditions: COPD (OR 1.52, 95% CI 1.46-1.59), asthma (OR 1.14, 95% CI 1.08-1.20), diabetes mellitus short-term complications (OR 1.42, 95% CI 1.33-1.50), and uncontrolled diabetes (OR 2.34, 95% CI 2.20-2.50). In contrast, schizophrenia was associated with decreased odds of hospitalization for congestive heart failure, diabetes mellitus long-term complications, angina without procedure, and diabetes related lower-extremity amputation. Schizophrenia was not associated with hospitalization for hypertension.

## Discussion

We conducted an analysis of 15,275,337 medical and surgical hospitalizations in the US from 2003–2008 to evaluate the association between schizophrenia and ACS hospitalizations. Schizophrenia was associated with a 17% increased odds of any ACS hospitalization, lower than the 83% increased odds associated with schizophrenia in Li’s study of hospital discharges in New York State. The magnitude of the association between schizophrenia and ACS hospitalization in our nationwide US sample was slightly lower than the heightened risk, ranging from 20% to 48%, of ACS hospitalizations for African Americans versus whites in a study of hospitalizations in ten US states [35]. In our study, schizophrenia was associated with increased odds of acute ACS hospitalization. The association between schizophrenia and chronic ACS hospitalization differed across the specific ACS indicators, suggesting that schizophrenia is associated with increased odds of hospitalization for some chronic PQIs characterized by short-term exacerbations.

We found a consistently positive association between schizophrenia and hospitalization for all acute ACS conditions. In addition, schizophrenia was associated with increased odds of hospitalization for chronic ACS conditions characterized by short-term exacerbations, including COPD, asthma, diabetes mellitus short-term complications, and uncontrolled diabetes. Like acute ACS conditions, these four chronic ACS conditions may require quick recognition and timely treatment in order to prevent hospitalization.

Persons with schizophrenia may experience barriers to timely primary care [20,21]. At the individual level, positive symptoms and cognitive impairments associated with schizophrenia may impede symptom recognition and delay treatment seeking [10,26]. Primary care providers receive little training regarding care for persons with schizophrenia, and several studies suggest that primary care physicians may misperceive physical symptoms as psychosomatic among patients with schizophrenia, [28,41] potentially delaying appropriate treatment for acute conditions that need timely care in order to avoid hospitalization.

Health systems factors may also contribute to the relationship between schizophrenia and increased odds of hospitalization for acute ACS conditions and chronic ACS conditions typified by short-term exacerbation. Poor access to care may prevent the prompt identification and treatment needed to prevent hospitalization for this group of ACS conditions; at least two studies suggest that persons with schizophrenia report delays in obtaining needed care [20,21]. One study found that outpatients with schizophrenia were three or more times as likely as their counterparts without schizophrenia to report that they had to wait too long to see a provider or could not get through on the phone or get an appointment in time [21]. Poor care coordination may also contribute to delays in primary care. Coordination between mental health specialists and primary care physicians may be inhibited by their positions within separate delivery systems, particularly if information technology systems are not integrated and primary care physicians are unable to access specialists’ records or vice versa [29,42].

Schizophrenia was associated with decreased likelihood of hospitalization for long-term diabetes complications and diabetes lower extremity amputation, conditions characterized by slow deterioration over time. While delayed primary care may lead to hospitalization for acute or short-term chronic ACS conditions, overall high frequency of provider visits [20,22] among persons with schizophrenia may increase the likelihood that providers identify and treat long-term chronic conditions that progress slowly.

Schizophrenia was also associated with decreased odds of hospitalization for CHF and angina after adjustment. Both of these conditions are characterized by symptoms, such as fatigue and swelling (CHF) and chest, back, or shoulder pain (angina) that physicians could be misperceived as psychosomatic among persons with schizophrenia [28]. It is also possible that persons with schizophrenia seek hospital care for these conditions less frequently than adults without schizophrenia, perhaps because they are less likely to recognize the symptoms or perceive need for treatment.

### Limitations

Our study has several limitations. The NIS data does not link hospitalizations to individuals, so there may have been more than one hospitalization per person. While ACS hospitalizations have been shown to be associated with poor access to high quality primary care at the population level, [32] this is only one possible cause of ACS hospitalizations. The ACS hospitalization indicators were designed to signal potential areas of poor access to high quality care that need further investigation; while we have suggested possible explanations for the patterns observed, the indicators are not designed to identify underlying causes and our national data may mask differences by region or health system. Our results therefore suggest overall patterns in the association between schizophrenia and potentially preventable hospitalizations in the US. Measurement error, resulting from unidentified or unreported diagnoses of schizophrenia, PQI indicators, or medical comorbidities may bias results. Residual confounding from medical illness not reported during hospitalization for patients with schizophrenia could help explain the association between schizophrenia and ACS hospitalization, and our administrative database was unable to capture measures of disease severity or lifestyle factors, such as smoking, that may increase susceptibility to ACS hospitalizations. Nonetheless, our study was the first to report an association between schizophrenia and ACS hospitalizations in a nationally representative US sample, as well as the first to describe differences in this association across various acute and chronic ACS conditions.

## Conclusions

Schizophrenia is associated with increased likelihood of hospitalization for acute and some chronic ACS conditions characterized by short-term exacerbations, suggesting poor access to timely and effective primary care for these conditions in the United States. Additional research is needed to determine factors within health systems that contribute to the increased odds of acute ACS hospitalization in schizophrenia. Potentially modifiable barriers to coordination between mental health and primary care providers should be examined.

## Competing interests

The authors declare that they have no competing interests.

## Authors’ contributions

All authors (EK, EM, DF, and GD): 1) have made substantial contributions to conception and design, or acquisition of data, or analysis and interpretation of data; 2) have been involved in drafting the manuscript or revising it critically for important intellectual content; and 3) have given final approval of the version to be published.

## Pre-publication history

The pre-publication history for this paper can be accessed here:

http://www.biomedcentral.com/1471-244X/13/37/prepub
